# Phenotypic and Molecular Characterization of Hypervirulent and Multidrug-Resistant *Acinetobacter baumannii* Isolated from ICU Respiratory Infections

**DOI:** 10.1155/2024/9670708

**Published:** 2024-09-18

**Authors:** Jinjun Qiu, Peng Zhu, Kailash Wagh, Neha singh, Shaowei Dong

**Affiliations:** ^1^ Shenzhen Pingshan District People's Hospital Pingshan Hospital Southern Medical University, Shenzhen 518110, China; ^2^ Clinical Laboratory Shenzhen Pingshan District People's Hospital Pingshan Hospital Southern Medical University, Shenzhen 518110, China; ^3^ Department of Microbiology Dr Ulhas Patil Medical College and Hospital, Jalgaon, Maharashtra, India; ^4^ Department of Microbiology Pandit Jawahar Lal Nehru Memorial Medical College, Raipur, Chhattisgarh, India; ^5^ Department of Haematology and Oncology Shenzhen Children's Hospital, 7019 Yitian Road Futian, Shenzhen, China; ^6^ Paediatric Research Centre Shenzhen Children's Hospital, 7019 Yitian Road Futian, Shenzhen, China

## Abstract

The emergence of hypervirulent strains of *Acinetobacter baumannii* poses a significant threat in intensive care units (ICU). This study aimed to molecularly characterize hypervirulent *A. baumannii* strains isolated from ICU patients with respiratory infections. Six strains were isolated from ICU patients over one month. Isolates were identified by phenotypic characterization biochemical properties and 16s RNA sequencing. Antibiotic susceptibility testing was conducted followed by resistance genes detection by PCR. MLST, and PFGE were employed to analyse clonal relationships among strains. Plasmid replicon typing and plasmid transmission frequencies were determined. The isolated strains exhibited diverse clinical manifestations, including acute respiratory distress syndrome (ARDS). Antibiotic susceptibility testing revealed multidrug-resistance phenotype. Molecular analysis revealed a complex genetic landscape of antibiotic resistance genes, including ESBLs and carbapenemases, as well as virulence genes such as *omp*A, *csu*E, and *exo*S. The multiple sequence types indicating genetic diversity among the strains as ST1512, ST622, and ST149 (each type two isolates). Plasmid characterization revealed the presence of diverse replicon types associated with multidrug resistance. This study provides comprehensive insights into the phenotypic, molecular, and epidemiological characteristics of hypervirulent *A. baumannii* outbark in ICU.

## 1. Introduction

*Acinetobacter baumannii* (*A. baumannii*), a Gram-negative bacterium, has emerged as a nosocomial pathogen worldwide [1], posing a threat to public health due to its ability to develop resistance to multiple antibiotics [2]. A systematic review on regional variations and trends in antimicrobial susceptibility of *Acinetobacter baumannii* indicated that the prevalence of this pathogen in hospital infections varies significantly across different regions: 0.7% in the US, 1.6% in Europe, 1.9% in Latin America, 2.5% in Africa, 3.6% in Asia, and 4.6% in the Middle East. Reports have shown that the rates of multidrug resistance (MDR) in *Acinetobacter baumannii* vary significantly by region: between 77% and 87% in Africa, Asia, and Latin America; 47% in North America; and over 93% in the Middle East and Europe. Additionally, it has been observed that MDR rates are higher in ICU settings compared to conventional hospital wards [3]. Its prevalence and impact are particularly pronounced in intensive care units (ICU), where vulnerable patients with compromised immune systems, invasive medical devices, and prolonged hospital stays are at increased risk of infection [4]. The relentless adaptability and persistence of *A. baumannii* within healthcare settings, coupled with its propensity for multidrug resistance (MDR), have led to a growing concern among healthcare professionals and researchers alike [5]. ICUs are hotspot for *A. baumannii* infections due to several factors [4]. Critically ill patients admitted to ICUs often require invasive procedures, such as mechanical ventilation, urinary catheterization, and central venous catheter insertion, which provide entry points for opportunistic pathogens like *A. baumannii* [6]. Additionally, the compromised immune status of ICU patients, resulting from underlying medical conditions or immunosuppressive therapies, renders them more susceptible to infections, including those caused by *A. baumannii* [7]. Furthermore, prolonged hospitalization and frequent exposure to broad-spectrum antibiotics in the ICU create a favourable environment for the selection and proliferation of drug-resistant strains of *A. baumannii* [8]. Patients infected with drug-resistant *A. baumannii* often experience poorer clinical outcomes compared to those infected with susceptible strains [9]. Treatment options become limited, leading to increased morbidity, mortality, and healthcare costs. Moreover, the spread of resistant strains within healthcare facilities exacerbates the challenge of infection control and prevention, posing a significant burden on healthcare systems worldwide [10]. Therefore, understanding the mechanisms underlying the emergence, transmission, and persistence of drug-resistant *A. baumannii* is imperative for developing effective strategies to combat its spread and improve patient outcomes [11]. This study investigates the genomic dynamics and transmission patterns of hypervirulent and multidrug-resistant strains of *A. baumannii* to elucidate the molecular mechanisms driving their emergence and dissemination. Through comprehensive genomic analysis and resistance gene detection, we aim to identify genetic determinants associated with virulence and antimicrobial resistance in *A. baumannii* strains isolated from ICU patients. The primary objectives of this research are threefold. Firstly, to characterize the genomic architecture and diversity of hypervirulent and multidrug-resistant strains circulating in ICUs. Secondly, to investigate the transmission dynamics of resistance genes within and between healthcare facilities, shedding light on the routes of dissemination and potential reservoirs of resistant strains. Lastly, to assess the clinical impact of drug-resistant *A. baumannii* infections in ICU patients, including factors influencing patient outcomes and healthcare resource utilization. We aim to inform evidence-based strategies for infection control, antimicrobial stewardship, and patient management, ultimately mitigating the impact of this formidable nosocomial pathogen on public health. By comparing our findings with data from other nations facing similar challenges, such as those in Europe, Asia, and the Americas, we can better understand the global landscape of *A. baumannii* infections. This comparative perspective can inform international strategies for infection control, antimicrobial stewardship, and policy-making. Ultimately, this research aims to contribute to worldwide efforts to curb the spread of this formidable nosocomial pathogen and improve patient care outcomes across diverse healthcare settings.

## 2. Materials and Methods

This study was conducted according to the guidelines of the Declaration of Helsinki and approved by the Institutional Ethics Committee of the Medical Institute which complies with international ethical standards. Informed consent was obtained from patients or their legal representatives before sample collection. Confidentiality of patient information was strictly maintained throughout the study.

### 2.1. Isolation and Identification

Samples were collected from six patients admitted to the ICU in June 2023 presenting with suspected *A. baumannii* infections. Specimens included blood (*n* = 4) and urine (*n* = 2) samples, which were obtained aseptically following standard clinical procedures. Blood samples were inoculated onto Columbia blood agar supplemented with 5% sheep blood (Oxoid Ltd., UK) and MacConkey agar (Oxoid Ltd., UK). Urine samples were streaked onto MacConkey agar. All media were supplemented with cephalosporins group-cefotaxime (4 *μ*g/mL), aminoglycosides group-Gentamicin (10 *μ*g/mL) and carbapenems group-meropenem (2 *μ*g/mL) antibiotics to inhibit the growth of other bacterial species and enhance the selective isolation of resistant *A. baumannii*. All Inoculated plates were then incubated aerobically at 37°C for 24 hrs. Growth characteristics, including colony morphology, colour, and haemolysis patterns, were carefully observed. Isolates presumptively identified as *A. baumannii* based on colony morphology were subjected to further confirmation using a combination of molecular techniques-including automated identification with the VITEK®2 Compact system followed by 16S rRNA gene amplification and sequencing [12]. The isolates were subjected to automated identification using the VITEK®2 Compact system according to the manufacturer's instructions. This system utilizes biochemical tests and advanced algorithms for accurate species identification. Furthermore, genomic DNA was extracted from pure cultures of presumptive *A. baumannii* isolates using a commercial DNA extraction kit (QIAamp DNA Mini Kit, Qiagen, Germany). The 16S rRNA gene was amplified by polymerase chain reaction (PCR) using universal primers, followed by Sanger sequencing. Sequence analysis was performed using the bioinformatics tool BLAST (Basic Local Alignment Search Tool) available through the National Centre for Biotechnology Information (NCBI)-https://blast.ncbi.nlm.nih.gov/Blast.cgi to species confirmation.

### 2.2. Antimicrobial Susceptibility Test

Antimicrobial susceptibility testing (AST) was conducted using the VITEK®2 Compact system with AST-N335 card for the following antibiotics: Penicillin/*β*-lactamase inhibitor combination-piperacillin/tazobactam, cephalosporins group-cefoperazone/sulbactam, cefepime, monobactam-aztreonam, carbapenems group-imipenem, meropenem, aminoglycosides-tobramycin, fluoroquinolones group-ciprofloxacin, levofloxacin, tetracycline group-doxycycline, minocycline, trimethoprim/sulfamethoxazole, tigecycline, and colistin. The VITEK®2 system provided an automated interpretation of antimicrobial susceptibility results, classifying isolates as susceptible, intermediate, or resistant to each tested antimicrobial agent based on the Clinical and Laboratory Standards Institute (CLSI) guideline M100-S30 [13]. The control strain ATCC 25922 was used.

### 2.3. ESBLs and MBLs Detection

Extended-spectrum beta-lactamases (ESBLs) and Metallo-Beta-Lactamases (MBLs) detection using the combined disc diffusion test [14]. The tests were performed using cefotaxime/clavulanate and ceftazidime/clavulanate for ESBLs, and imipenem/EDTA for MBLs, following CLSI guidelines. The formation of a characteristic inhibition zone indicated the presence of these enzymes.

### 2.4. Resistance and Virulence Gene Detection

Based on the AST result followed the detection of antibiotic resistance genes and virulence genes, including biofilm-related genes, primers and PCR conditions were adopted from previously published studies with established protocols [15, 16]. The resistance genes include Imparting resistance to *β*-lactam antibiotics-bla_OXA_,  bla_EBC,_ bla_DHA,_ bla_CIT,_ bla_CMY,_ bla_OXA−48,_ bla_FOX,_ bla_TEM_,  bla_CTX−*M*_,  bla_SHV_,  bla_TEM_,  bla_PER_, Mediating resistance to aminoglycosides-*aac* (6′) -Ib, *aph* (3′)-Ia, Fluoroquinolones resistance *qnr*A, *qnr*B, *qnr*S, *par*C, *gyr*A, tetracycline group *tet* (A), *tet* (B), *tet* (C), and colistin resistance *mcr-1*, *pmr*A, B. While virulence genes are *omp*A is associated with outer membrane protein A, *csu*E, *bap* and *exo*S encoding the *Exo*S effector protein. Following PCR amplification, gel electrophoresis was employed to visualize the amplified DNA fragments, confirming the presence or absence of the target genes in the isolates. Positive controls from our laboratory were included, and DH_10_B DNA was used as a negative control to validate the PCR assays.

### 2.5. MLST and PFGE

Multilocus sequence type (MLST) and Pulse-Filed Gel Electrophoresis (PFGE) were performed to analyse the clonal relationship between the six isolates. The extracted DNA was assessed for quality and purity using UV-visible spectrophotometry. A spectrophotometer measures the absorbance of DNA at wavelengths of 260 nm and 280 nm. The ratio of absorbance at A260/A280 is used to assess DNA purity, with values around 1.8 considered indicative of pure DNA. The Pasteur scheme for MLST was used for *A. baumannii*, with PCR amplification of seven housekeeping genes: *glt*A, *gyr*B, *gdh*B, *rec*A, *cpn*60, *gpi*, and *rpo*D. Primer sequences are specific to each gene locus and PCR conditions are adopted from https://pubmlst.org/primers-used-mlst-acinetobacter-baumannii-complex-pasteur-scheme for amplification. The PCR products are purified and sequenced bidirectionally using Sanger sequencing platforms. The resulting sequences are trimmed and aligned to generate a consensus sequence for each housekeeping gene locus. The sequences of the seven housekeeping gene loci are compared to allele sequences in the MLST database for *A. baumannii*. Each unique allele at a specific locus was assigned. The combination of alleles at the seven loci defines the sequence type (ST) of the isolate. The MLST profiles, including the allelic profiles and sequence types, were analysed using existing MLST databases. This allows for the assignment of sequence types and the identification of clonal complexes and phylogenetic relationships among isolates.

For PFGE high-resolution agarose gel was prepared with a 2% agarose in 0.5 X Tris-borate-EDTA (TBE) buffer. The digested DNA by 50 U of *XbaI* samples are loaded into the wells of the agarose gel. Electrophoresis is performed using a PFGE system with 0.5 × TBE buffer at 14°C for 18 h at a gradient of 6 V/cm, with a pulse angle of 120°. The DNA fragments are separated based on their size using alternating pulses of electric field strength. Following electrophoresis, the gel is stained with a suitable DNA stain, such as ethidium bromide. The DNA fragments are visualized under UV light or using a gel documentation system. Dendrogram analysis was performed using Bio-Numerics version 7.6 to generate a phylogenetic tree based on the PFGE patterns to determine the genetic relatedness of the isolates [17]. Dendrogram analysis using appropriate clustering algorithms was performed to generate a phylogenetic tree based on the PFGE patterns.

### 2.6. Conjugation and Plasmid Replicon Type

Conjugation experiments were conducted to investigate the horizontal gene transfer of antibiotic resistance genes. Streptomycin-resistant *Escherichia coli* strain C_600_ was used as the recipient strain, while our six isolates as donor strains, harbouring the plasmid with antibiotic-resistance genes, were cultured separately. The protocol for meeting essays was adopted from our collaborative lab [18]. Antibiotic selection was performed using cefotaxime at a concentration of 4 *μ*g/mL to selectively inhibit the growth of the recipient strain and allow for the transfer and expression of the plasmid-borne resistance genes. Conjugation efficiency and the transfer of antibiotic resistance were assessed through selective plating and subsequent antibiotic susceptibility testing. The plasmid replicon types of the conjugates were characterized using a PCR-based replicon typing method. Primers targeting specific replicon sequences associated with various plasmid incompatibility groups were obtained from the online database of the Plasmid Multilocus Sequence Typing (pMLST) website (https://pubmlst.org/plasmid/). The plasmid replicon typing for *A. baumannii* was performed using a method validated by Bertini et al. [19]. PCR products were analysed by agarose gel electrophoresis, and the presence of specific replicon types was determined based on the size of the amplified fragments. This approach provided valuable insights into the diversity of plasmids involved in the transfer of antibiotic resistance genes among bacterial populations.

## 3. Results

A total of six strains of *A. baumannii* were isolated from clinical specimens, comprising blood samples (*n* = 4) and urine samples (*n* = 2), collected from patients in the ICU. The median age of the patients was 60 years, with a standard deviation of ±5 years (SD = 5). Among the patients, five were male, while one was female. Clinical manifestations observed in the patients included acute respiratory distress syndrome (*n* = 3), traumatic brain injury (*n* = 1), gastrointestinal bleeding (*n* = 1), and sepsis infection (*n* = 1) (S-1).

### 3.1. Phenotypic and Biochemical Characterization

**Figure 1:**
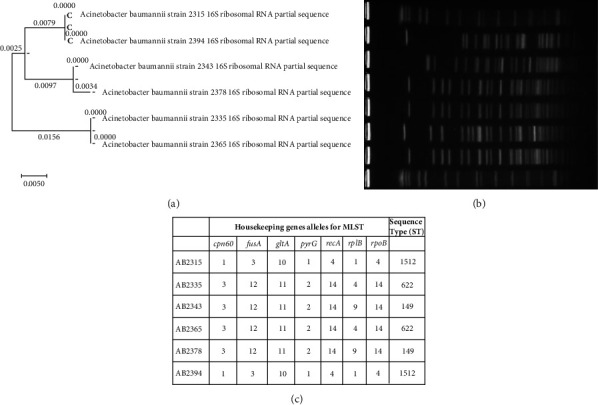
Phylogenetic analysis of hypervirulent *Acinetobacter baumannii* isolates based on 16s rRNA sequencing.

The isolated strains were characterized based on their phenotypic and biochemical properties. Nonhaemolytic, pale, and smooth appearance on MacConkey agar. The phylogenetic tree analysis based on 16S rRNA sequencing depicted the relationship of the isolated strains with previously published clones, providing insights into their genetic relatedness and evolutionary history (Figure 1(a)).

### 3.2. Antibiotic Susceptibility

**Figure 2:**
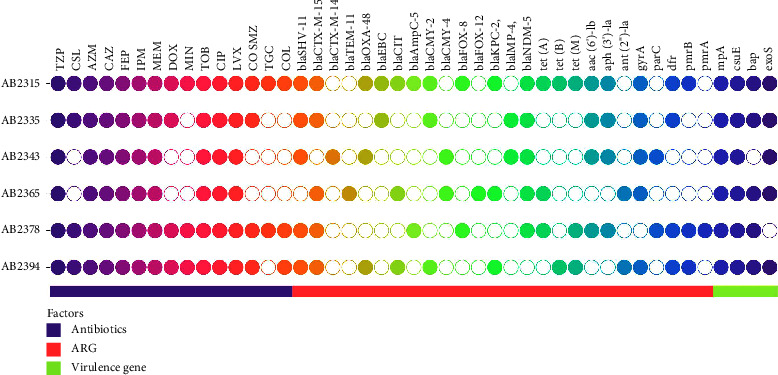
Comprehensive characterization of hypervirulent *Acinetobacter baumannii* isolates: antibiotic susceptibility, resistance genes, and virulence factors.

The antibiotic susceptibility profile of hypervirulent strains revealed widespread resistance across all strains. Complete resistance (100%) was observed for piperacillin/tazobactam, cefoperazone/sulbactam, imipenem, meropenem, ciprofloxacin, levofloxacin, and tigecycline. Partial susceptibility was noted for aztreonam, ceftazidime, and cefepime, with 83% of strains showing resistance and 17% demonstrating susceptibility. Similarly, minocycline exhibited resistance in 83% of strains, with 17% displaying susceptibility. Tobramycin, trimethoprim/sulfamethoxazole, and colistin also showed resistance in 83% of strains, with 17% demonstrating susceptibility. Additionally, all hypervirulent *Acinetobacter baumannii* strains (*n* = 6) produced both ESBLs and MBLs (Figure 2). These findings underscore the high prevalence of resistance mechanisms in hypervirulent *A. baumannii* strains and emphasize the critical need for robust antimicrobial stewardship practices to combat multidrug-resistant infections in clinical settings.

### 3.3. Molecular Detection of Resistance and Virulence Genes

The analysis of resistance genes across sir strains revealed a diverse distribution of genetic determinants associated with antibiotic resistance. Among the tested strains (*n* = 6), the ESBLs encoding genes bla_SHV−1_ was found in 100% of strains (*n* = 6), bla_CTX−*M*−15_ [83.33% (*n* = 5)], bla_CTX−*M*−14_ [16.66% (*n* = 1)], bla_OXA−48_,  bla_CMY−2_, and bla_CIT_ [50% (*n* = 3)] each, bla_EBC,_ bla_AmpC−5_,  bla_FOX−8_ and bla_CMY−2_ [33.33% (*n* = 2)] each while bla_FOX−12_ in one strain. Additionally, carbapenem resistance encoding genes bla_NDM‐5_ was found in 100% of strains (*n* = 6), bla_KPC−2_ [50% (*n* = 3)] and bla_IMP−4_ [33.33%(*n* = 2)]. Other notable findings include the detection of the tetracycline resistance encoding gene *tet* (A) gene in 33% of strains (*n* = 2), and the *tet* (B) [16.7% (*n* = 1)]. Fluoroquinolones genes *gyr*A in 100% (*n* = 6), and dfr in 83% of strains (*n* = 5). Furthermore, aminoglycosides resistance genes such as *aac* (6′)-Ib, *aph* (3′)-Ia, were detected in 50% of strains (*n* = 3) (Figure 2). These findings underscore the diverse genetic repertoire of antibiotic resistance mechanisms among hypervirulent *A. baumannii* strains, highlighting the complex nature of multidrug resistance in these isolates. In addition to resistance gene detection, the analysis also included the identification of virulence genes in hypervirulent strains. The results indicated the presence of multiple virulence genes across the strains. Among the tested strains (*n* = 6), the *ompA* gene was detected in all strains, representing a prevalence of 100%. The *csu*E gene was also found in all strains, indicating its ubiquitous presence among the isolates. The bap gene was detected in 83.3% of strains (*n* = 5), while the *exo*S gene was identified in 66.7% of strains (*n* = 4). Notably, the combination of *omp*A, *csu*E, and *exo*S genes was observed in all strains, suggesting a consistent genetic profile associated with virulence across the isolates. The presence of bap was observed in 50% of strains (*n* = 3), while the combination of *omp*A, *csu*E, and bap genes was detected in 33.3% of strains (*n* = 2) (Figure 2). These findings highlight the diverse repertoire of virulence determinants among hypervirulent *Acinetobacter baumannii* strains, underscoring the potential for enhanced pathogenicity and virulence-associated traits in these isolates.

### 3.4. Clonal Relation

Based on the results of MLST, the strains were classified into sequence types (STs) as follows: ST1512, ST622, and ST149. Among these, ST1512 and ST149 were each represented once, while ST622 was observed twice, accounting for 33.33% of the total strains (*n* = 2). Strains were identified with repetitive STs, indicating potential clonality within the sample set. Allelic combinations are depicted in Figure 1(c). For Pulse-Field Gel Electrophoresis (PFGE), two distinct digestion patterns were observed, as illustrated in Figure 1(b). These patterns indicate significant genetic diversity among the strains. Despite sharing some sequence types based on MLST, the observed diversity in PFGE patterns suggests variations in genomic backgrounds or evolutionary lineages among the strains. This diversity underscores the importance of utilizing multiple typing methods to comprehensively characterize bacterial isolates. The distinct PFGE patterns highlight the genetic heterogeneity among the hypervirulent strains studied, emphasizing the need for a multifaceted approach to understanding their genetic makeup and potential virulence factors.

### 3.5. Plasmid Characteristics

**Figure 3:**
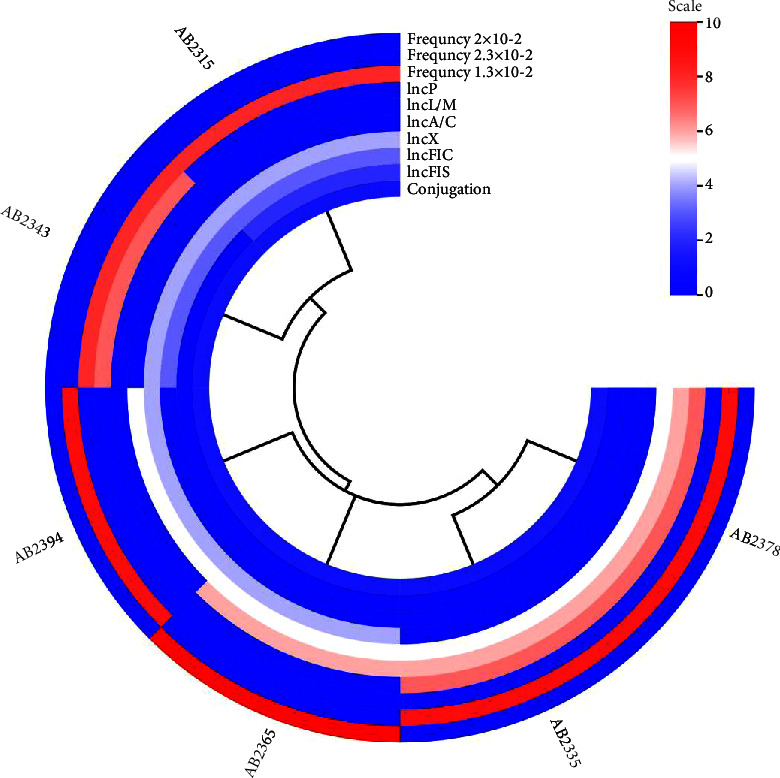
Plasmid characteristics of the conjugants were determined based on the replicon types and plasmid transmission frequencies observed.

The plasmid characteristics of the conjugants were determined based on the replicon types and plasmid transmission frequencies observed. The identified replicon types and their respective frequencies provide insights into the diversity of plasmids present in the hypervirulent *A. baumannii* strains studied (Figure 3). The predominant replicon types observed among the conjugants were IncA/C, IncL/M, and IncP, with a frequency of 2.3 × 10^−2^. These plasmids are commonly associated with multidrug resistance and may play a significant role in the dissemination of antibiotic-resistance genes among bacterial populations. Additionally, IncFIC, IncFIA, IncP, and IncX replicon types were identified in conjugants at a frequency of 1.3 × 10^−2^. Overall, the presence of diverse replicon types highlights the complex nature of plasmid-mediated gene transfer and emphasizes the importance of understanding plasmid characteristics in the dissemination of antibiotic resistance and virulence traits among bacterial populations.

## 4. Discussion

The isolation of six hypervirulent strains of *A. baumannii* from clinical specimens, particularly from patients in the ICU, underscores the importance of understanding the epidemiology and pathogenic potential of this bacterium in healthcare settings. The median age of the patients (60 years) and the observed clinical manifestations, such as acute respiratory distress syndrome, traumatic brain injury, gastrointestinal bleeding, and sepsis infection, reflect the severity and diversity of conditions associated with *A. baumannii* infections in critically ill patients. These results highlight the urgent need for effective infection control measures and targeted therapeutic interventions to mitigate the impact of *A. baumannii* infections, especially in vulnerable patient populations [20]. Phenotypic and biochemical characterization of the isolated strains provided valuable insights into their morphological and physiological traits. The characteristic appearance of colonies on MacConkey's agar was consistent with previous descriptions of *A. baumannii* colonies. Furthermore, phylogenetic analysis based on 16S rRNA sequencing elucidated the genetic relatedness and evolutionary history of the isolated strains, revealing their phylogenetic placement relative to previously published strains. This comparative analysis aids in understanding the genetic diversity and evolutionary relationships among hypervirulent *A. baumannii* strains, contributing to our broader understanding of their pathogenesis and spread [21]. The antibiotic susceptibility profile revealed alarming levels of multidrug resistance, posing significant challenges for clinical management and infection control. The widespread resistance observed against various antibiotics underscores the urgent need for novel antimicrobial strategies to combat *A. baumannii* infections. Notably, the detection of ESBLs and MBLs in all strains further exacerbates the issue of antimicrobial resistance, highlighting the urgent need for enhanced surveillance and stewardship efforts to limit the dissemination of resistant strains [22]. Comparisons with published literature suggest that the observed resistance patterns align with global trends of increasing antimicrobial resistance in *A. baumannii* [23]. These findings underscore the critical role of ongoing surveillance and research efforts to monitor antimicrobial resistance profiles and inform therapeutic strategies. The molecular detection of resistance and virulence genes revealed a complex genetic landscape characterized by diverse antibiotic resistance mechanisms and virulence determinants. Among the strains, a wide array of resistance genes associated with ESBLs and carbapenemases were identified. Notably, genes encoding ESBLs, such as bla_SHV−1_ and bla_CTX−*M*−15_, and the co-existence of carbapenem resistance genes, including bla_NDM−5_ and bla_KPC−2_, were detected. Many studies reported the coexistence of both ESBL and MBL-encoding genes globally, highlighting the urgent need for novel therapeutic strategies [24]. Our findings are significant as they suggest a potential outbreak in the single ICU ward, as routine examination suggested. Similar outbreaks have been reported in Switzerland [25] and USA [26]. Other resistance genes, such as *tet* (A), *tet* (B), *gyr*A, and *dfr*, were also observed, further complicating treatment strategies [27]. Furthermore, the analysis revealed the presence of multiple virulence genes across the strains, underscoring their potential for enhanced pathogenicity. The consistent presence of virulence gene combinations, such as *mp*A, *csu*E, and *exo*S, suggests a conserved genetic profile associated with virulence among the isolates. Similarly, the detection of virulence genes, including *omp*A, *csu*E, *bap*, and *exo*S, underscores the diverse virulence potential of hypervirulent *A. baumannii* strains, contributing to their pathogenicity and clinical impact [28]. The clonal relationship analysis based on MLST revealed three sequence types among the strains, suggesting genetic relatedness within the sample set. Additionally, PFGE analysis identified two distinct genetic patterns, indicating significant genetic diversity among the strains. These findings highlight the importance of employing multiple typing methods to comprehensively characterize *A. baumannii* isolates and understand their genetic makeup and potential virulence factors. The plasmid characteristics of the conjugants were examined to elucidate the role of plasmids in disseminating antibiotic resistance and virulence determinants. The analysis revealed a diverse array of replicon types among the conjugants, reflecting the complexity of plasmid-mediated gene transfer in *A. baumannii*. Predominant replicon types, such as IncA/C, IncL/M, IncFIC, IncFIA, IncP, IncX, and IncP, were observed, indicating their potential involvement in multidrug resistance. These replicon types are commonly associated with the carriage of resistance genes and have been implicated in the dissemination of antibiotic resistance among bacterial populations. The presence of diverse replicon types underscores the dynamic nature of plasmid-mediated gene transfer and highlights the importance of understanding plasmid characteristics in the spread of antibiotic resistance and virulence traits [29]. Comparisons with published literature indicate both similarities and differences in plasmid characteristics among *A. baumannii* strains [30]. While certain replicon types, such as IncA/C and IncL/M, are commonly observed across different studies, the prevalence of specific replicon combinations may vary depending on geographic location, clinical settings, and strain diversity. The identification of diverse replicon types underscores the significance of plasmids in shaping the antibiotic resistance and virulence profiles of hypervirulent *A. baumannii* strains. Further research into plasmid dynamics and the interplay between plasmids and chromosomal elements is warranted to better understand the mechanisms driving the dissemination of antibiotic resistance and virulence genes in *A. baumannii* populations. While our study provides valuable insights into the resistance and virulence profiles of hypervirulent *A. baumannii* strains, several limitations should be acknowledged. Firstly, the AST was performed using broth dilution methods, which may not fully capture the complexity of the resistance mechanisms present in these strains. Whole-genome sequencing would provide a more comprehensive understanding of novel drug-resistance genes or virulence factors and aid in elucidating the evolutionary dynamics of *A. baumannii* populations. Additionally, the sample size in our study was relatively small, and short period limiting the generalizability of our findings. Future studies with larger sample sizes and broader geographic representation are needed to validate our results and provide a more comprehensive understanding of hypervirulent *A. baumannii* strains' resistance and virulence profiles.

## 5. Conclusion

Our study highlights the alarming levels of multidrug resistance and the diverse genetic landscape of antibiotic resistance and virulence determinants among *A. baumannii*. These findings underscore the need for enhanced surveillance, infection control measures, and the development of novel therapeutic strategies to combat multidrug-resistant infections caused by such clones in clinical settings. While our research provides valuable insights, there are still unexplored areas that warrant further investigation. Specifically, focus on elucidating the precise mechanisms driving antibiotic resistance and virulence. Additionally, it is crucial to explore novel therapeutic targets and develop innovative intervention strategies to address the issue of multidrug resistance. Emphasizing these future directions will help in developing more effective treatments and containment strategies for these challenging infections.

## Data Availability

The data used in this study are available from the corresponding authors upon reasonable request.

## Supplementary Materials

Supplementary Materials Demographic and Clinical Characteristics of Patients with Hypervirulent *Acinetobacter baumannii* Infection in the ICU.
