# Supplementary material for: Phenotypic and Molecular Characterization of Hypervirulent and Multidrug-Resistant Acinetobacter baumannii Isolated from ICU Respiratory Infections
Source: Can J Infect Dis Med Microbiol. 2024 Sep 18;2024:9670708. doi: 10.1155/2024/9670708 (PMC11424856; doi:10.1155/2024/9670708)
Supplement: Supplementary Materials — Demographic and Clinical Characteristics of Patients with Hypervirulent Acinetobacter baumannii Infection in the ICU. [file 9670708.f1.docx]

***Supplementary material***

**S-1: Demographic and Clinical Characteristics of Patients with Hypervirulent *Acinetobacter baumannii* Infection in the ICU**

| Sr No | ID | Gender | Age (Yrs.) | Specimen | Isolate Date | Ward | Clinical Manifestation |
| --- | --- | --- | --- | --- | --- | --- | --- |
| 1 | AB2315 | Male | 65 | blood | 15-Jun-23 | ICU-1 | Acute Respiratory Distress Syndrome (ARDS) |
| 2 | AB2335 | Male | 48 | blood | 16-Jun-23 | ICU-1 | Traumatic Brain Injury |
| 3 | AB2343 | Male | 62 | urine | 18-Jun-23 | ICU-1 | Acute Respiratory Distress Syndrome (ARDS) |
| 4 | AB2365 | Male | 67 | blood | 18-Jun-23 | ICU-1 | Acute Respiratory Distress Syndrome (ARDS) |
| 5 | AB2378 | female | 55 | urine | 20-Jun-23 | ICU-1 | Gastrointestinal Bleeding |
| 6 | AB2394 | Male | 59 | blood | 20-Jun-23 | ICU-1 | Sepsis Infection |
